# Molecular Basis of Glia–ECM Interplay in Central Nervous System Homeostasis and Plasticity

**DOI:** 10.3390/cells15141268

**Published:** 2026-07-15

**Authors:** Lanlan Wang, Xinyuan Ma, Qinghong Zeng, Mi Liu, Zenghui Yue, Rong Hu

**Affiliations:** College of Acupuncture-Moxibustion, Tuina and Rehabilitation, Hunan University of Chinese Medicine, Changsha 410208, China; m15397919822@163.com (L.W.); xyma13761212326@163.com (X.M.);

**Keywords:** extracellular matrix, glial cells, neuroinflammation, mechanotransduction, central nervous system homeostasis

## Abstract

**Highlights:**

**What are the main findings?**

- The extracellular matrix and glial cells form a dynamic bidirectional signaling network that governs central nervous system (CNS) homeostasis and plasticity.
- Mechanical cues, biochemical signaling, and glia-driven extracellular matrix (ECM) remodeling cooperatively regulate neuroinflammation, synaptic stability, and myelination.

**What are the implications of the main findings?**

- Dysregulation of the glia-ECM interactome contributes to neurodegeneration, demyelination, and regenerative failure in neurological disorders.
- Targeting ECM mechanics, matrix composition, and glial phenotypes offers an integrated therapeutic framework for neural repair and translational neuroscience.

**Abstract:**

Interactions between glial cells and the central nervous system (CNS) extracellular matrix (ECM) maintain neural homeostasis and plasticity. In contrast to traditional reviews focusing on biochemistry, this work summarizes the structural and mechanical properties of major ECM subtypes, and illustrates how mechanical and biochemical signals jointly regulate glial activity via key mechanosensitive pathways. Glia in turn dynamically remodel ECM, forming a reciprocal regulatory network. This paper also analyzes characteristic mechanical disorders in Alzheimer’s disease, multiple sclerosis and traumatic brain injury, and addresses targeted therapeutic strategies. Current research challenges and emerging interdisciplinary directions are also discussed. This review provides a valuable reference for exploring neurological disease mechanisms and developing new therapies.

## 1. Introduction

The central nervous system (CNS) relies on a well-organized neurovascular unit to maintain its physiological function and homeostasis. This functional complex is composed of neurons, glial cells, the extracellular matrix (ECM), endothelial cells and pericytes. Rather than a static structural scaffold, the ECM serves as an active signaling platform [1,2]. Its biochemical and physical properties are tightly regulated throughout embryonic development, adulthood and aging [3]. In addition to providing structural support for neural tissues, the ECM delivers essential instructive cues that modulate cellular behaviors, synaptic transmission and post-injury tissue repair [4,5], which has been well documented in numerous previous studies.

Central nervous system ECM comprises two major structural forms: a widespread diffuse interstitial matrix and highly organized specializations, including vascular basement membranes and perineuronal nets (PNNs) surrounding subsets of neurons [4]. This structural versatility underpins a functional spectrum spanning from guiding neurogenesis and axonal pathfinding during development to stabilizing synapses, modulating neuronal excitability, and regulating classic developmental critical period plasticity, and also mediating the reactivation of plasticity windows in the adult mature brain [6,7]. The physical properties of the ECM, including stiffness (elastic modulus), viscoelasticity and surface topography, are not only basic physical characteristics of brain tissue but also core regulatory factors governing cell fate and tissue homeostasis.

Over the past five years, CNS research has witnessed a clear shift in research paradigms. Early work largely focused on biochemical signals and neuroimmune responses. Today, CNS mechanobiology has become a fast-growing field. Mechanical cues cooperate with biochemical pathways to regulate glia-ECM crosstalk, and this mechanochemical coupling has now become a major research focus in neuroscience. For example, Strat et al. recently published a study on how astrocytes sense, integrate, and respond to biomechanical signals through mechanosensitive receptors, intracellular signaling pathways, and downstream cellular responses [8]. The dynamic composition and organization of the ECM are therefore fundamental to its role as a key regulator of CNS plasticity and function.

Astrocytes, microglia and oligodendrocytes are not just inert structural components within the ECM microenvironment. Instead, they act as key architects, sensors and remodelers of this network [3,9,10,11]. Astrocytes secrete large amounts of hyaluronan (HA) and chondroitin sulfate proteoglycans (CSPGs). Their processes wrap around synapses, forming the so-called tetrapartite synapse, contributing to the tetrapartite synapse model in which ECM actively functions with presynaptic, postsynaptic, and astroglial elements [12,13,14]. Microglia, the resident immune cells, continuously survey and remodel the ECM through phagocytic clearance and protease secretion, playing a decisive role in synaptic pruning and extracellular space homeostasis [15]. This continuous, bidirectional crosstalk between glia and the ECM forms a core regulatory axis for CNS stability and adaptability.

This delicate regulatory system easily breaks down under pathological conditions. In traumatic injury, stroke, Alzheimer’s disease and other neuroinflammatory disorders, ECM composition and mechanical properties change abnormally, accompanied by glial overactivation [15,16]. Reactive astrocytes overexpress inhibitory CSPGs, which leads to the formation of growth-limiting glial scars [17]. Simultaneously, dysfunctional microglia may fail to properly clear or may excessively degrade ECM components like PNNs, leading to synaptic instability and loss of neuroprotection [15]. This cascading disorder of the glia-ECM interactome ultimately induces a series of pathological phenotypes, including excessive neuroinflammation, synaptic loss, impaired neural plasticity and failure of nerve tissue regeneration, which are the common pathological features of most refractory neurological diseases.

This review summarizes the latest mechanistic advances in glia-ECM interactions. We first describe the composition, structure and physiological biomechanical features of the CNS-ECM. Next, we discuss how aberrant mechanical and biochemical signals alter glial phenotypes, and how glia in turn remodel the surrounding ECM. We then link glia-ECM dysfunction to biomechanics-related neurological disorders, and finally outline novel therapies targeting this mechanochemical axis, covering the modulation of ECM mechanical properties, optimization of ECM biochemical composition and reprogramming of glial phenotypes. Collectively, these perspectives support the concept that the glia-ECM interactome functions as an integrated mechanochemical signaling axis, rather than a set of independent structural components and inflammatory processes.

## 2. Structural and Biomechanical Features of ECM

**Figure 1:**
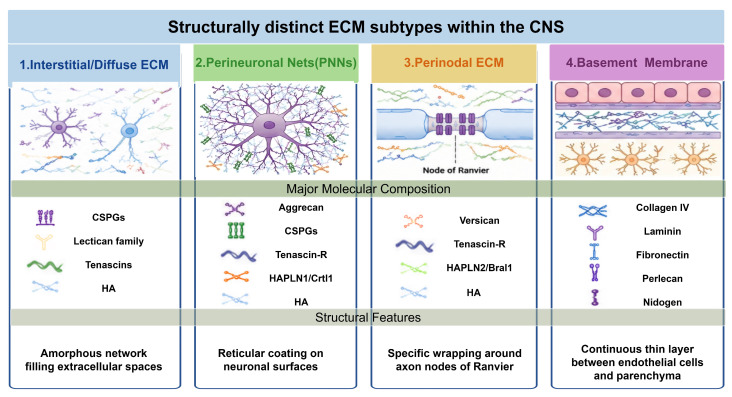
Schematic diagram illustrating four structurally distinct extracellular matrix (ECM) subtypes within the central nervous system. (1) Diffuse interstitial ECM forms loose networks filling gaps between parenchymal neurons and glia. (2) Dense perineuronal nets (PNNs) wrap neuronal somata and proximal dendrites. (3) Specialized perinodal ECM specifically surrounds the nodes of Ranvier along myelinated axons. (4) Continuous basement membrane lies at the boundary between vascular endothelial cells and brain parenchyma to support blood–brain barrier function.

The ECM is a sophisticated three-dimensional network in the CNS, composed of structural proteins (e.g., collagen, elastin, fibronectin, laminin) and polysaccharides (e.g., HA, proteoglycans, and glycosaminoglycans). Based on structural and compositional characteristics, the CNS ECM can be classified into four functionally distinct subtypes, a classification that reveals the structural basis for its functional diversity and provides an important theoretical framework for understanding the molecular mechanisms of glia-ECM-neuron interactions under physiological and pathological conditions [18,19,20,21]. Their composition and functional characteristics are summarized in Table 1 and Figure 1.

### 2.1. Interstitial/Diffuse ECM

The interstitial ECM is an extracellular network filling gaps between parenchymal neural cells that are not tightly associated in basement membranes or perineuronal nets; indeed, it comprises a dense network of proteoglycans, HA, tenascins and link proteins, as well as relatively small amounts of fibrous proteins (that is, collagens and elastin), glycosaminoglycans, and adhesive glycoproteins (that is, laminins and fibronectin), which provides structural support between cells [15,22].

This diffuse ECM network occupies the extracellular spaces distributed across the CNS parenchyma, contributes to signal transmission and structural support, directly affects the geometry and charge distribution of the extracellular space, influencing ion diffusion, neurotransmitter concentration gradients, and water balance, thereby providing a suitable microenvironmental buffer for neural network activity [3,23].

### 2.2. Perineuronal Nets (PNNs)

As dense reticular structures, perineuronal nets (PNNs) wrap around the soma, dendrites and proximal axons of specific neuronal populations (mainly GABAergic inhibitory neurons expressing parvalbumin and Cornu Ammonis area 2 (CA2) pyramidal neurons) [12,23,24,25]. It is mainly composed of aggregated proteoglycans, hyaluronan, cartilage link protein1 (Crtl1/HAPLN1), tenascin-R (TN-R), negatively charged glycosaminoglycans, and a high content of CSPGs with high-density negative charges on the side chains, forming a highly organized reticular structure [12,23,24,25,26]. PNNs play a central role in stabilizing synaptic structures, regulating the ionic microenvironment (especially Na^+^, K^+^, Ca^2+^ concentrations), and limiting the contact range of astrocytic processes, thereby maintaining excitatory/inhibitory balance, protecting neurons from oxidative stress, and regulating critical period plasticity [25].

### 2.3. Perinodal ECM

Another specialized ECM subtype forms around nodes of Ranvier along myelinated axons, known as the perinodal ECM [23]. Its composition is distinct from PNNs and includes versican, hyaluronan, tenascin-R, and hyaluronan and proteoglycan link protein 2 (HAPLN2). This structure affects the clustering and function of nodal sodium channels (e.g., Nav1.6), directly influencing action potential generation and conduction efficiency [27,28].

### 2.4. Basement Membrane

The basement membrane constitutes a key part of the blood–brain barrier (BBB). It lies at the boundary between vascular endothelial cells and brain parenchyma [22]. It primarily consists of type IV collagen, laminin, fibronectin, nidogen, and perlecan [22,29]. This specialized structure not only performs filtration and barrier functions but also regulates cell polarity, differentiation, and ion channel protein anchoring via integrin-mediated signal transduction, laying the foundation for the functional integrity of the neurovascular unit [30].

### 2.5. Detection and Experimental Manipulation Technologies for CNS Biomechanics

ECM endows the central nervous system with a distinct physical architecture. The biophysical properties of this microenvironment-primarily its stiffness (elastic modulus), viscoelasticity, and topography-constitute a fundamental layer of information that is actively sensed by glial cells [31]. This process, known as mechanotransduction, enables glial cells to convert physical cues into biochemical signals and functional responses [32]. A set of detection and manipulation technologies has been widely applied to clarify the mechanistic links between ECM mechanics and glial behaviors.

A set of mainstream techniques for CNS mechanobiology includes mechanical detection tools and experimental intervention approaches, supporting the quantitative analysis of tissue mechanics and the construction of simulated pathological microenvironments. Atomic force microscopy (AFM) is a popular tool for evaluating the mechanical properties of biological materials (cells and tissues) at high resolution [33,34]. With nanoscale resolution, it is widely adopted by research groups worldwide. Combined with immunofluorescence staining, AFM can measure the stiffness of individual glial cells, glial scars and perineuronal nets, and further link mechanical phenotypes to molecular characteristics [35]. Magnetic resonance elastography (MRE) is a non-invasive in vivo imaging technique that captures shear wave propagation in brain tissue to quantify its shear stiffness at both global and regional levels, including focal lesions [36]. Rheometers are widely used to characterize the viscoelastic properties of brain-mimicking hydrogels and engineered biomaterials, enabling precise tuning of their stiffness and viscoelastic behavior to match those of native brain tissue [37,38,39].

For experimental manipulation, stiffness-tunable polyacrylamide and hyaluronan composite hydrogels are the most common platforms to construct in vitro models that simulate physiological and pathological mechanical microenvironments [32,40]. An injectable composite hydrogel has been developed and demonstrated in vivo in a spinal cord injury model to promote neural regeneration and functional recovery [41]. Beyond static scaffolds, emerging mechanically active biomaterial systems, including stimuli-responsive hydrogels and 3D bioprinted constructs, can generate controllable mechanical cues to modulate pathological microenvironments and support tissue regeneration. These approaches represent a rapidly evolving direction in regenerative tissue engineering, including applications in neural repair [42,43].

### 2.6. Neurological Disorders with Altered CNS Biomechanics

The physiological biomechanical properties of the CNS as the fundamental guarantee for maintaining glial homeostasis and normal neural function [44,45]. Once tissue stiffness exceeds the pathological threshold of 5 kPa or undergoes abnormal softening, the mechanosensing capabilities of astrocytes, microglia and oligodendrocyte lineage cells will be disordered, triggering a cascade of pathological changes such as excessive neuroinflammation, demyelination, and synaptic dysfunction. A variety of common neurological diseases are accompanied by typical biomechanical abnormalities, and mechanical dysregulation has been proven to be an early initiating factor of lesions rather than a secondary consequence [46].

#### 2.6.1. Alzheimer’s Disease (AD)

Alzheimer’s disease, a common age-related neurodegenerative disorder, is pathologically featured by β-amyloid plaques and neurofibrillary tangles. Its brain tissue presents heterogeneous mechanical properties: the global brain softens, while the microenvironment surrounding amyloid-beta (Aβ) plaques becomes extremely stiff. Clinical MRE and in vitro AFM tests have confirmed this dual mechanical change, which is closely associated with disease progression [47]. Clinical MRE measurements demonstrated a significant reduction in global brain stiffness in Alzheimer’s disease patients, with median shear modulus decreasing to approximately 2.20 kPa compared with cognitively normal controls (~2.32–2.37 kPa), indicating global mechanical softening associated with disease progression [47]. Collectively, AFM-based studies demonstrate that Aβ plaque-associated microenvironments exhibit significantly increased local stiffness compared with surrounding brain tissue, forming mechanically distinct niches that are sensed by microglial Piezo type mechanosensitive ion channel component 1 (Piezo1) and regulate microglial activation [48,49].

#### 2.6.2. Multiple Sclerosis (MS)

Multiple sclerosis is an autoimmune demyelinating disease targeting CNS white matter, and its lesions show distinct dynamic biomechanical changes along with demyelination and remyelination processes [50]. In the acute phase of MS, inflammatory cell infiltration and initial myelin loss lead to transient softening of focal white matter lesions, and the viscoelasticity of local tissue is significantly impaired [50]. As the disease progresses to the chronic progressive stage, continuous demyelination, abnormal deposition of fibronectin and CSPGs in lesions, and persistent activation of glia collectively increase the stiffness of damaged white matter. The stiffness of chronic demyelinating plaques stably exceeds 5 kPa, which is far beyond the physiological mechanical threshold of normal white matter [51]. Clinical MRE follow-up studies found that partial recovery of tissue stiffness can be observed in patients with relieved symptoms, which is accompanied by partial reconstruction of myelin and ECM, proving the close correlation between mechanical properties and disease activity of MS lesions [52].

#### 2.6.3. Traumatic Brain Injury (TBI)

Traumatic brain injury caused by external mechanical force triggers immediate acute tissue damage and long-term glial scar formation, with staged biomechanical changes throughout the pathological process [53]. In the acute phase after injury, external force directly tears the original ordered structure of interstitial ECM and PNNs, resulting in widespread disorder of tissue viscoelasticity without fixed stiffness values [54]. Different from fibrotic scars in peripheral tissues, the glial scar formed in the subacute and chronic stages of TBI has a unique mechanical characteristic: the core area of the scar is extremely soft (elastic modulus about 50 Pa), while the peripheral transition zone of the scar forms a high-stiffness microenvironment of 5–8 kPa [55]. Multiple AFM-based studies have demonstrated that traumatic brain injury induces significant alterations in brain tissue mechanics, characterized by a heterogeneous stiffness distribution between the lesion core and the surrounding perilesional region [55,56]. These biomechanical changes are closely associated with glial scar formation and extracellular matrix remodeling.

## 3. Core Mechanisms of Glia–ECM Interactions

**Figure 2:**
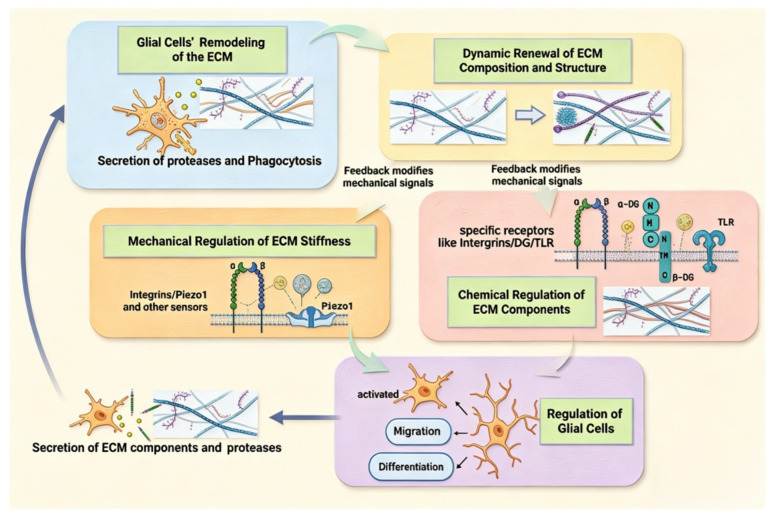
Core Mechanisms of Glia-ECM Interactions.

ECM and glia form a complex bidirectional network. This network sustains CNS homeostasis and modulates pathological changes such as neurodegeneration and neural repair [18,19,20,21]. This bidirectional regulation is primarily manifested in three aspects: mechanical signal transduction, chemical signal recognition, and dynamic remodeling. As illustrated in Figure 2, the ECM directs and regulates glial cells through mechanical and chemical signals, while glial cells actively remodel the dynamic balance of the ECM through enzymatic degradation, phagocytic clearance, and synthesis/secretion.

### 3.1. Instructive Regulation of Glia by ECM via Mechanical Signals

**Figure 3:**
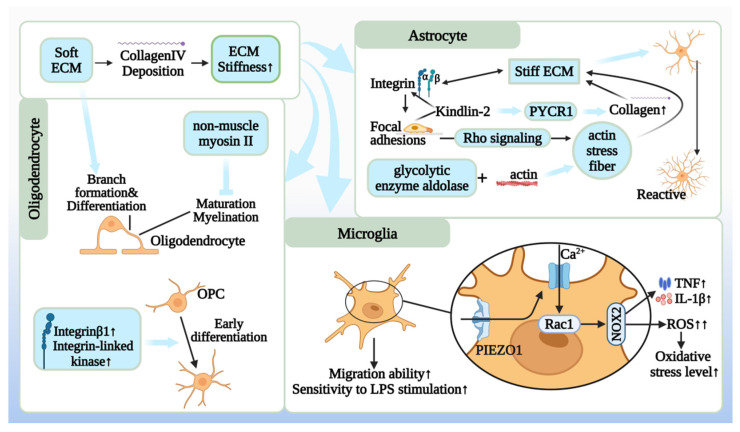
Mechanical signal mechanisms. Mechanical signals regulate the phenotypic plasticity and behaviors of microglia, astrocytes, and oligodendrocytes. Microglia: In response to increased ECM stiffness caused by collagen IV deposition, PIEZO1 channels open, allowing a rapid influx of Ca^2+^. It triggers downstream pro-inflammatory pathways and leads to TNFα and IL-1β secretion and ROS generation, thereby increasing oxidative stress. Astrocytes: Astrocytes perceive mechanical cues primarily through integrin-based focal adhesions, leading to Rho-associated protein kinase activation and assembly of robust actin stress fibers. The focal adhesion protein, Kindlin-2, assists in integrin activation and then it binds to PYCR1, leading to collagen deposition and finally increasing ECM rigidity. The glycolytic enzyme aldolase binds intracellular F-actin to remodel the cytoskeleton, and further increases ECM rigidity indirectly. It renders astrocytes adopt the reactive morphology. Oligodendrocytes: Soft substrates support branching and differentiation of oligodendrocyte precursor cells (OPCs). By contrast, non-muscle myosin II (NMII) blocks their maturation and myelination, while the β1-integrin/ILK pathway drives early differentiation. Abbreviation note: Neuronal anoikis refers to the apoptosis of neurons induced by the loss of adhesion to the extracellular matrix. OPC, Oligodendrocyte Precursor Cell; ECM, Extracellular Matrix; Piezo1, Piezo type mechanosensitive ion channel component 1; NOX2, NADPH Oxidase 2; ROS, Reactive Oxygen Species; TNF-α, Tumor Necrosis Factor-α; IL-1β, Interleukin-1β; ILK, Integrin-Linked Kinase; NMII, Non-muscle Myosin II.

Under physiological conditions, the CNS maintains a remarkably soft and pliable environment, with grey and white matter exhibiting similar elastic moduli in the range of hundreds of Pascals (Pa), which keeps astrocytes, microglia and OPCs in a quiescent state [53]. Pathological events, however, frequently trigger aberrant matrix stiffening, often by an order of magnitude or more [53]. Deviations from this parameter range will directly trigger abnormal activation of glial cells and disrupt glia-ECM homeostasis [40,57]. The mechanical regulatory axis governing glial phenotypes is systematically summarized in Figure 3.

#### 3.1.1. Microglia: Immunomechanosensation of Substrate Mechanical Properties

Immunomechanosensation refers to the ability of microglia to sense the mechanical stiffness of the surrounding microenvironment and convert mechanical cues into immune activation signals. As the primary immune sentinels of the CNS, microglia are exquisitely sensitive to changes in the mechanical properties of their substrate, which profoundly influences their activation state, motility, and inflammatory output.

##### Mechanosensitive Channels as Stiffness Decoders

Microglia sense mechanical changes mainly through the mechanosensitive channel PIEZO1 [58,59]. When ECM stiffness rises, PIEZO1 opens and triggers rapid calcium influx [59,60,61]. This stiffness-induced Ca^2+^ signaling acts as a master switch, triggering downstream pro-inflammatory pathways [40]. It leads to the reactive oxygen species (ROS) and proinflammatory cytokines including TNFα and IL-1β, promoting classical proinflammatory microglial polarization [40,59]. This pathway establishes a mechanistic link between pathological ECM stiffening and proinflammatory immune activation [40].

Controlled in vitro studies using polyacrylamide hydrogels of tunable stiffness have quantified this relationship [62]. Microglia cultured on stiff substrates (mimicking disease, e.g., 8–10 kPa) display significantly enhanced migratory capacity, elevated oxidative stress, and a heightened inflammatory response to immune challenges like lipopolysaccharide (LPS), compared to those on soft, brain-mimetic substrates (~0.5 kPa) [40]. Microglia rapidly sense and respond to this local stiffening, altering their morphology and clustering at the mechanically distinct boundary, demonstrating their continuous adaptation to the physical microenvironment during injury responses [63].

#### 3.1.2. Astrocytes: Morphological and Phenotypic Plasticity Driven by Mechanics

Astrocytes, the versatile supporters of neuronal function and blood–brain barrier integrity, undergo significant morphological and functional changes in response to ECM stiffness, a process central to the formation of the glial scar.

##### Integrin-Mediated Sensing and Cytoskeletal Reorganization

Astrocytes perceive mechanical cues largely through integrin-based focal adhesions. Upon engagement with a stiff ECM, integrin clusters recruit and activate downstream signaling proteins, leading to the assembly of robust actin stress fibers via Rho-associated protein kinase activation [57]. Kindlin-2 typically functions as a focal adhesion protein to assist in integrin activation. However, on a rigid substrate, it translocates to the mitochondria, binds to pyrroline-5-carboxylate reductase 1 (PYCR1), and enhances its stability. PYCR1 is the key enzyme for proline synthesis, and proline is a major component of collagen, which means that it will lead to collagen deposition, further stiffening of the ECM, and creation of a vicious cycle [32]. In addition, proteins can perform other non-enzymatic functions in addition to their original catalytic functions. For example, the glycolytic enzyme aldolase, in addition to its catalytic function in glycolysis, can directly bind to intracellular F-actin and promote actin bundle assembly. This remodeling of the astrocytic cytoskeleton alters the cellular mechanical phenotype, and further drives excessive synthesis and deposition of ECM components via downstream signaling, ultimately elevating extracellular matrix rigidity [32]. This force-dependent cytoskeletal remodeling translates into dramatic morphological alterations. On stiff 2D substrates (~2000 Pa), astrocytes adopt a spread, flattened, and highly branched reactive morphology, with increased surface area and complex process arborization [53]. In contrast, on soft substrates (~200 Pa), they remain smaller and more quiescent [53].

#### 3.1.3. Oligodendrocyte Lineage: Mechanoregulation of Myelination

The development and regenerative capacity of the myelinating oligodendrocyte lineage are intimately regulated by the physical properties of their microenvironment, influencing every stage from precursor cell fate to mature myelin sheath formation [64].

Oligodendrocyte precursor cells (OPCs) sense and respond to ECM stiffness through β1-integrin-mediated adhesion [65,66]. On rigid substrates mimicking early injury or fibrotic scars, OPCs preferentially proliferate and undergo limited early differentiation but are ultimately inhibited from maturing fully into myelinating oligodendrocytes [66,67]. This stiffness-activated β1-integrin/ILK (Integrin-Linked Kinase) pathway sustains a progenitor-like state [66,67].

Conversely, a soft, brain-like mechanical environment (~100–500 Pa) is strongly permissive for terminal differentiation and myelination. This permissive signal involves the downregulation of stiffness-sensing pathways. A key mediator is non-muscle myosin II (NMII), a molecular motor that generates cytoskeletal contractility [68]. On stiff matrices, sustained NMII activity maintains high intracellular tension, which inhibits the extensive branching and membrane expansion required for myelination [69]. Pharmacological or genetic inhibition of NMII on stiff substrates can rescue oligodendrocyte differentiation and myelin basic protein expression, demonstrating that mechanical signals are dominant over many soluble factors in controlling this cell fate decision [69].

### 3.2. Specific Regulation of Glia by ECM Biochemical Components

**Figure 4:**
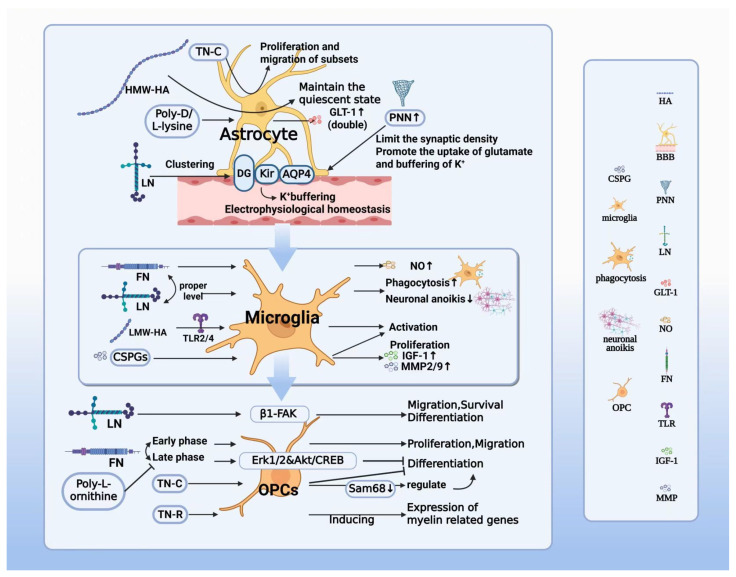
Chemical signal mechanisms. The chemical molecules in ECM regulate glial behaviors. Upward and downward arrows (↑ and ↓) indicate increased and decreased levels, activities, or functional responses of the indicated molecules, ECM components, or cellular processes, respectively. Abbreviations: PNN, Perineuronal Net; BBB, Blood–Brain Barrier; GLT-1, Glutamate Transporter 1; TN-C, Tenascin-C; TN-R, Tenascin-R; HMW-HA, High-Molecular-Weight Hyaluronan; LN, Laminin; FN, Fibronectin; CSPGs, Chondroitin Sulfate Proteoglycans; TLR, Toll-Like Receptor; IGF-1, Insulin-Like Growth Factor 1; MMP, Matrix Metalloproteinase; OPC, Oligodendrocyte Precursor Cell.

Beyond mechanical forces, the ECM functions as a rich and dynamic repository of biochemical information. Distinct molecular components within the ECM, including glycoproteins, proteoglycans, and glycosaminoglycans, act as highly specific ligands that bind to complementary receptors on glial cell surfaces [70]. This interaction initiates precise intracellular signaling cascades that dictate glial cell identity, state, and function. This section delineates how key ECM molecules exert cell-type-specific regulation over astrocytes, microglia, and oligodendrocytes, shaping their roles in CNS homeostasis and disease. The key biochemical signaling events between ECM components and glia are depicted in Figure 4.

#### 3.2.1. Regulation of Astrocyte Function by ECM Cues

Astrocytes, essential for synaptic support and ionic balance, are exquisitely tuned by the surrounding ECM chemistry, which governs their morphology, electrophysiological properties, and reactivity.

##### Structural and Functional Polarization via Basement Membrane Components

Specialized ECM structures, particularly the basement membrane, are critical for astrocyte polarization and function. The glycoprotein laminin, a core component, interacts with the dystroglycan complex on astrocyte endfeet [71,72]. This interaction is indispensable for the targeted clustering of key channels, including the inwardly rectifying potassium channel 4.1 (Kir4.1) and the water channel aquaporin-4 (AQP4) [71]. This molecular anchoring at the gliovascular interface is fundamental for potassium spatial buffering and water homeostasis, ensuring efficient clearance of neuronal activity byproducts [73,74]. Disruption of the laminin-dystroglycan link, as seen in some dystroglycanopathies, leads to mislocalization of these channels, impaired K^+^ buffering, and increased seizure susceptibility [75]. Furthermore, ECM coatings such as poly-ornithine or poly-D/L-lysine in vitro have been shown to double the expression of the glutamate transporter 1 (GLT-1; excitatory amino acid transporter 2, EAAT2) compared to non-coated surfaces, highlighting how substrate chemistry directly regulates astrocytic capacity for neurotransmitter clearance, a process vital for preventing excitotoxicity [76].

##### Modulation of Reactivity and Plasticity by Diffuse Matrix and PNNs

Interstitial matrix and PNN components regulate astrocyte reactivity and synaptic function. High-molecular-weight HA (HMW-HA) stabilizes the quiescent astrocyte phenotype under physiological conditions [77]. In contrast, injury-induced enzymatic breakdown of HMW-HA into low-molecular-weight fragments releases this suppression, contributing to reactive astrogliosis, proliferation, and scar formation [78]. The TN-C glycoprotein, upregulated in injury, creates a permissive environment that supports the migration and proliferation of specific astrocyte subsets during scar evolution [79]. Moreover, PNNs, composed largely of CSPGs like aggrecan, physically constrain astrocytic processes, shaping their territorial domains and fine-tuning their contact with synapses [24]. This structural confinement is crucial for forming the tripartite synapse and regulates astrocyte-mediated synaptic plasticity [24]. Degradation of PNNs leads to excessive astrocytic ensheathment of synapses, altering synaptic transmission and contributing to pathologies such as epilepsy [24].

#### 3.2.2. Regulation of Microglia Phenotype and Function by ECM Signals

As the CNS’s primary immune cells, microglia are highly responsive to ECM-derived molecular patterns, which dictate their adhesion, migration, activation state, and inflammatory output [19,20].

##### Adhesion-Dependent Activation and Phagocytic Priming

Microglial interaction with the ECM via integrin receptors (e.g., αvβ3 and αvβ5) is a prerequisite for their surveillance and effector functions [80]. Adhesion to fibronectin (FN), mediated by α5β1 integrin, provides a strong activation signal, promoting a pro-inflammatory phenotype characterized by the release of nitric oxide and pro-inflammatory cytokines [81]. Maintaining balanced levels of these adhesive substrates is thus crucial; while FN can drive inflammation, appropriate LN signaling supports microglial survival and can enhance their phagocytic clearance of cellular debris, a key reparative function [82].

##### Sensing Damage and Driving Inflammation via Degradation Products

Fragmentation products which act as damage-associated signaling cues, which reflect the pathological status of CNS tissue and initiate downstream signaling cascades. The most characterized example is HA. In its native, high-molecular-weight form, HA is anti-inflammatory [83]. Upon injury or disease, HA is cleaved into low-molecular-weight hyaluronan (LMW-HA) [78,83]. These LMW-HA fragments are potent agonists for microglial Toll-like receptors 2 and 4 (TLR2/4) and cluster of differentiation 44 (CD44), triggering a robust pro-inflammatory response, nuclear factor kappa B (NF-κB) activation, and the production of cytokines like TNF-α and IL-1β [23]. Similarly, CSPGs, when presented as a substrate, can induce microglial activation, proliferation, and the secretion of factors like insulin-like growth factor-1 (IGF-1) and matrix-degrading enzymes (MMP-2, MMP-9) [23,84]. This positions microglia as key interpreters of ECM integrity, switching from homeostatic surveillance to active immune response upon detecting these “danger signals.”

#### 3.2.3. Regulation of Oligodendrocyte Lineage Dynamics by ECM

The development and regenerative capacity of the myelinating oligodendrocyte lineage are stringently controlled by a sequence of permissive and inhibitory ECM signals.

##### Stage-Specific Cues for Development and Remyelination

The influence of ECM molecules on oligodendrocyte precursor cells (OPCs) and mature oligodendrocytes is highly stage-dependent. Laminin, signaling predominantly through β1-integrin and focal adhesion kinase (FAK), provides a consistently positive signal, promoting OPC migration, survival, and differentiation into myelinating oligodendrocytes throughout development and in remyelination contexts [29]. In stark contrast, fibronectin (FN) exerts a biphasic effect. Its transient, early expression after demyelination is beneficial, aiding in OPC recruitment to the lesion site [85]. However, its persistent accumulation, often as insoluble aggregates, becomes powerfully inhibitory [85]. Chronic FN signaling, mediated by α5β1 integrin, activates intracellular pathways (e.g., suppressing protein kinase B (Akt)/cAMP response element-binding protein (CREB) that block OPC differentiation, contributing to remyelination failure in diseases like multiple sclerosis [85].

##### Modulation by Other Matrix Components

Other ECM molecules fine-tune this process. Tenascin-C inhibits OPC migration but can promote differentiation via RNA-binding protein Sam68, whereas tenascin-R supports myelination by stabilizing axon-oligodendrocyte interactions and inducing myelin gene expression [86]. The CSPG-rich environment of chronic glial scars forms a potent chemical barrier, inhibiting OPC differentiation and process extension. Therapeutic interventions, such as applying poly-L-ornithine, can neutralize the inhibitory effects of FN, demonstrating the potential to reprogram the ECM from inhibitory to permissive for repair [87].

Specific ECM molecules precisely regulate glial behavior and function by binding to specific cell surface receptors. Table 2 summarizes the effects and molecular mechanisms of major ECM Componentt on the three glial cell types. This specificity underscores that the ECM is not a monolithic entity but a mosaic of localized signals. The functional outcome in the CNS is the integrated sum of these simultaneous, cell-specific conversations. Disruption of this precise molecular dialogue, through abnormal deposition, degradation, or altered receptor expression, is a common driver of glial dysfunction across neurological disorders.

### 3.3. Remodeling of the ECM by Glial Cells

**Figure 5:**
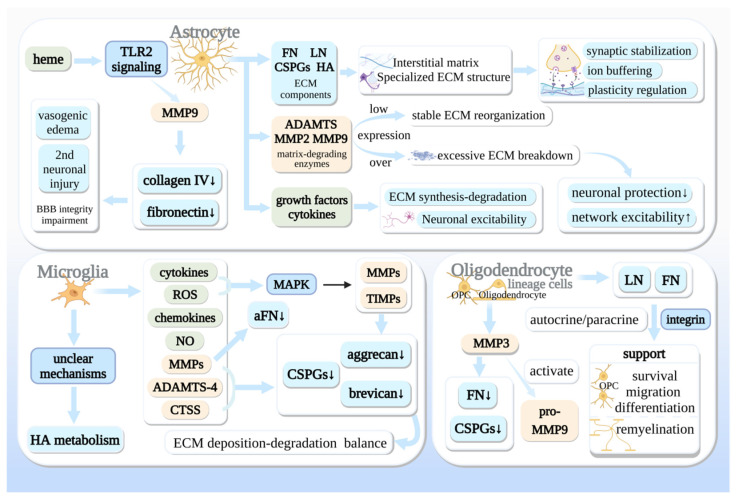
ECM remodeling mechanisms. Reactive Astrocytes: Upon activation by heme and TLR2 signaling, reactive astrocytes secrete thrombospondin, tenascin, MMPs, ADAMTS proteases, and tPA, which alter ECM composition, degrade PNNs and thin interstitial matrix, ultimately leading to network hyperexcitability. This process disrupts BBB integrity, and drives glial scar formation. Over-secreted CSPGs form scar tissue, probably trigger inflammation and inhibit axonal sprouting and remyelination, impeding functional recovery. Astrocytes within PNNs also selectively express homeostatic proteins that support the clearance of ions and transmitters released from synapses. Microglia: Microglia exert phagocytosis and release specific proteases and chemokines, to modify the ECM landscape. The release of cytokines and ROS activates MAPK pathway, driving ECM remodeling; the release of MMPs and ADAMTS-4 to alter ECM deposition. However, the molecule mechanisms of their regulation of HA metabolism remain unclear. Oligodendrocytes: Oligodendrocytes provide support to synapses via LN and FN, secrete MMPs to modulate ECM and regulate demyelination, and regulate neuronal growth and migration. Upward and downward arrows (↑ and ↓) indicate increased and decreased levels of ECM components, molecules, or functional outcomes, respectively. Abbreviations: ECM, Extracellular Matrix; PNN, Perineuronal Net; BBB, Blood–Brain Barrier; TLR2, Toll-like Receptor 2; MMP, Matrix Metalloproteinase; TIMP, Tissue Inhibitor of Metalloproteinase; ADAMTS, A Disintegrin And Metalloproteinase with Thrombospondin Motifs; tPA, Tissue Plasminogen Activator; CSPG, Chondroitin Sulfate Proteoglycan; ROS, Reactive Oxygen Species; NO, Nitric Oxide; MAPK, Mitogen-Activated Protein Kinase; HA, hyaluronan; LN, Laminin; FN, Fibronectin.

**Figure 6:**
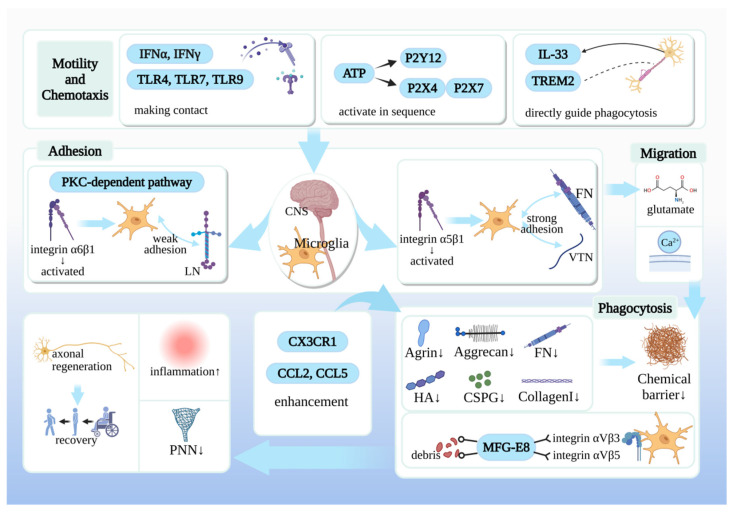
Phagocytosis And Clearing Mechanisms. Motility and chemotaxis: interferon-α (IFNα) and interferon-γ (IFNγ) and TLR4/TLR7/TLR9 mediate the initial contact between microglia and target sites. Extracellular ATP first triggers sequential receptor activation: Purinergic receptor P2Y12 (P2Y12) is activated to initiate microglial migratory function, followed by purinergic receptor P2X4 (P2X4) and purinergic receptor P2X7 (P2X7) activation to drive motility and chemotaxis. Additionally, neuronal IL-33 acts through TREM2 to directly guide microglial phagocytosis; Adhesion: Following chemotaxis, microglia establish adhesion via two distinct pathways. Weak adhesion is mediated by activated integrin α6β1, which binds to LN through a PKC-dependent pathway. Strong adhesion is mediated by activated integrin α5β1, which binds to FN and VTN; Migration: Glutamate signaling and Ca^2+^ influx drive directional migration of microglia toward the target site; Phagocytosis: MFG-E8 functions as a bridging molecule: it recognizes phosphatidylserine (PS) exposed on the membrane of apoptotic cells and debris via its two discoidin domains, and binds to microglial integrins αVβ3 and αVβ5 via its two EGF-like domains, thereby tethering debris and apoptotic cells to microglia and upregulating multiple receptors on microglia to promote myelin debris phagocytosis. C-X3-C motif chemokine receptor 1 (CX3CR1), together with chemokines such as CCL2 and CCL5, further enhances this phagocytic process. Upward and downward arrows (↑ and ↓) indicate increased and decreased levels of ECM components, inflammatory responses, or functional outcomes, respectively. Abbreviations: PNN, Perineuronal Net; IL-33, Interleukin-33; HA, Hyaluronic Acid; CNS, Central Nervous System; IFN, Interferon; TLR, Toll-like Receptor; TREM2, Triggering Receptor Expressed On Myeloid Cells 2; PKC, Protein Kinase C; LN, Laminin; FN, Fibronectin; VTN, Vitronectin; PS, Phosphatidylserine; EGF, Epidermal Growth Factor; CX3CR1, C-X3-C Motif Chemokine Receptor 1; CCL, C-C Motif Chemokine ligand.

Glia actively shape the CNS extracellular matrix through synthesis, proteolytic cleavage, and phagocytic clearance. These processes maintain CNS homeostasis and regulate tissue repair after injury or disease, as shown in Table 3. The balance between constructive and degradative activities [98] determines whether glia-ECM interactions support homeostasis or drive pathology. The overall ECM remodeling processes driven by reactive astrocytes, microglia, and oligodendrocytes are integrated in Figure 5. Microglial phagocytic and clearance mechanisms underlying ECM remodeling are detailed in Figure 6.

#### 3.3.1. Astrocytes: Key Regulators of ECM Structure and Stability

Astrocytes are primary producers and remodelers of CNS-ECM. They secrete proteases to restructure the matrix and modulate synaptic plasticity and stability [26], supporting neural repair. Astrocytes also secrete growth factors and cytokines that regulate ECM synthesis-degradation homeostasis, and influence neuronal excitability by adjusting ECM composition, ion channel distribution, and synaptic plasticity [99].

##### Synthesis and Assembly of the ECM Scaffold

Central nervous system ECM assembly is supported by both astrocytes and neurons, with astrocytes serving as prominent sources of many interstitial and injury-induced ECM components [100]. Astrocytes secrete fibronectin, laminin, CSPGs and HA [101,102], which maintain the interstitial matrix and contribute to specialized ECM structures. For PNN, astrocytes support matrix organization and integrity, but neurons are essential sources of aggrecan and other core PNN components required for net formation in multiple neuronal subtypes [24,103,104]. Astrocytic processes penetrate PNN to form tripartite synapses and participate in synaptic stabilization, ion buffering, and plasticity regulation [90,103].

Following injury, reactive astrocytes increase ECM production and participate in glial scar formation [105]. The glial scar is a multifunctional structure that initially contains inflammation, preserves blood–brain barrier integrity, and limits tissue damage [105,106]. Astrocyte-derived ECM within the scar forms a provisional matrix that supports residual cells and permits limited axonal sprouting [90]. Upregulated ECM remodelers including MMP9, a disintegrin and metalloproteinase with thrombospondin motifs 1 (ADAMTS1), and tissue plasminogen activator (tPA) can degrade PNNs around inhibitory neurons and reduce interstitial matrix density [107,108].

##### Proteolytic Remodeling and Its Consequences

Astrocytes secrete multiple proteases including MMP2, MMP9, and a disintegrin and metalloproteinase with thrombospondin motifs (ADAMTS) family members [107,108]. Under physiological conditions, low-level protease activity supports subtle ECM reorganization required for synaptic plasticity [109]. In pathological states, dysregulated protease release leads to excessive ECM breakdown.

After intracerebral hemorrhage, heme triggers TLR2 signaling in astrocytes and increases MMP9 expression [110]. Elevated MMP9 degrades collagen IV and fibronectin in the vascular basement membrane, impairing blood–brain barrier integrity and promoting vasogenic edema and secondary neuronal injury [111,112]. In epilepsy, excessive protease activity from astrocytes degrades PNN around inhibitory interneurons, reducing neuronal protection and increasing network excitability [26]. Maintaining protease-antiprotease balance is critical for preserving CNS structural and functional stability.

#### 3.3.2. Microglia: The Specialized Phagocytes and Precision Modulators

Microglia remodel the ECM mainly through phagocytic clearance and targeted protease release, acting as sensitive regulators of the extracellular space [15].

##### Phagocytic Clearance of ECM Debris

Microglia continuously phagocytose ECM fragments to maintain homeostasis and refine neural circuits during development. After injury or during neurodegeneration, microglia accumulate at lesion sites to clear ECM debris, myelin breakdown products, and inhibitory CSPGs, which can locally reduce barriers to regeneration [15]. Neuronal interleukin-33 promotes microglial engulfment of ECM components such as aggrecan at synaptic sites, supporting normal synaptic remodeling and plasticity [12,113]. Microglial depletion leads to abnormal accumulation of ECM molecules, confirming their nonredundant role in ongoing matrix clearance [114].

##### Targeted Enzymatic Degradation

Microglia secrete several proteases that directly modify ECM structure. Cathepsin S (CTSS) cleaves core PNN components including aggrecan and brevican, regulating PNN integrity and associated synaptic plasticity [23]. In pathological pain models, microglial CTSS in the spinal dorsal horn degrades PNN and contributes to circuit disinhibition and hypersensitivity [115]. Microglia also express a disintegrin and metalloproteinase with thrombospondin motifs 4 (ADAMTS4), which cleaves CSPGs at sites distinct from matrix metalloproteinases (MMPs). MMP2 and ADAMTS4 act synergistically to degrade brevican [23]. In retinal degeneration studies, microglia modulate ECM components closely associated with their processes, suggesting repopulating microglia might attenuate excessive ECM deposition [116].

MMPs, ADAMTS, and/or cathepsins are proposed as likely mechanistic candidates mediating microglial regulation of ECM and synaptic components, expressed directly by microglia or influenced by other microglial cytokine secretion [23]. Cytokines and reactive oxygen species from activated microglia stimulate mitogen-activated protein kinase (MAPK) signaling, enhancing MMP transcription and reducing tissue inhibitor of metalloproteinase (TIMP) activity, further amplifying ECM remodeling [23,117]. While these processes support debris clearance, excessive microglial protease activity can disrupt ECM structure and exacerbate tissue damage. Activated microglia produce cytokines, chemokines, NO, and ROS, driving disease progression [40,118]. Cytokines and ROS can activate MAPKs, enhancing MMP transcription and reducing TIMPs, leading to ECM remodeling [119,120]. Activated microglia directly produce MMPs cleaving aFn, while damaged microglia produce MMP3 [121,122].

#### 3.3.3. Oligodendrocytes: ECM Contributors in Myelination and Repair

Oligodendrocyte lineage cells modulate the ECM to support their own development, migration, differentiation, and myelination. Oligodendrocyte precursor cells (OPCs) and mature oligodendrocytes secrete laminin and other ECM components [123]. These molecules act in an autocrine/paracrine manner via integrin receptors to promote OPC survival, migration, and differentiation [123,124]. Oligodendrocyte-derived ECM thus creates a supportive microenvironment that facilitates myelination during development and remyelination after damage. Damaged or stressed oligodendrocytes secrete MMP3, which degrades inhibitory ECM components including aggregated fibronectin and CSPGs [122]. MMP3 can also activate pro-MMP9, amplifying local ECM degradation [125]. This protease-mediated remodeling helps OPCs penetrate inhibitory glial scar environments and access demyelinated axons, supporting regenerative myelination.

**Table 3:** Expression of CNS ECM components across cell types.

Components	Neurons	NSCs	Astrocytes (NA/RA)	OL Lineage (OL/Pre-OL/OPC)	Microglia
PNN and Lectican Family					
Aggrecan [14,126,127,128]	++	+	+/+	+/ /+	+
Brevican [126,127,129,130]	+		++/++	++/ /++	
Versican [14,126,127,129,130,131]	++	+	++/++	−/++/+	
Neurocan [14,126,127,129,130,132]	++	+	+/++	+/+/+	+
Phosphacan [127,129,130]	++		++/++	++/ /++	
HAPLN1/Crtl1 [126,127,129]	++				
HAPLN4/Bral2 [104,133]	++		−/−	−/ /	−
Adhesive and Matrix Glycoproteins					
Tenascin-C [101,126,127,134,135,136]	++	+	+/++		
Tenascin-R [104,126,127,129,131,134]	++		+/	++/++/++	
Lectican [126,127,128]			+/+	+/ /	
Fibronectin [126,130,137]			+/+		+
Collagen [130,137,138]		+	+/+		
Perlecan [13,137,139,140]	+		/+		+
Agrin [104,130]	++		+/		
MMPs [127,141,142]	++	+	++/++	+/+/+	++

Note: This table summarizes the expression levels of major extracellular matrix (ECM) components in different CNS cell types. ++ = strong expression; + = clear expression; − = no expression; blank = no available data. Abbreviation: NSCs = neural stem cells; NA = naive astrocytes; RA = reactive astrocytes; OL = oligodendrocyte; OPC = oligodendrocyte progenitor cell.

## 4. Therapeutic Strategies Targeting Glia–ECM Interactions

The growing mechanistic understanding of bidirectional glia-ECM interactions has catalyzed the development of multiple innovative therapeutic strategies targeting this axis. These approaches—centered on modulating ECM biophysical properties, normalizing its biochemical composition, or reprogramming glial phenotypes—provide promising new directions for treating neurological diseases.

### 4.1. Modulating ECM Mechanical Properties

Pathological alterations in the mechanical properties of the CNS microenvironment, particularly increased ECM stiffness, constitute a formidable barrier to neural regeneration [53]. These rigid mechanical cues directly drive the pathological activation of glial cells, exacerbating neuroinflammation and inhibiting repair. Consequently, therapeutic strategies aimed at normalizing the biophysical dialogue between cells and their matrix have emerged as a critical frontier. These approaches focus on both providing a permissive physical scaffold and pharmacologically interrupting aberrant mechanotransduction pathways.

#### 4.1.1. Engineering Biomimetic Hydrogels to Restore a Permissive Niche

A widely adopted strategy uses soft, biodegradable hydrogels to mimic the mechanical properties of healthy neural tissue [54]. These engineered matrices serve a dual purpose: they fill physical defects and provide a three-dimensional scaffold that actively instructs cellular behavior. For instance, implantation of hyaluronan-methylcellulose composite hydrogels has been shown to guide astrocytes and microglia toward anti-inflammatory, pro-repair phenotypes [143]. The field is rapidly advancing beyond static scaffolds towards dynamic and responsive hydrogel systems. A notable example is a reactive oxygen species ROS-triggered hyaluronan-based soft scaffold developed for ischemic stroke repair [144]. This hydrogel not only simulates the brain ECM to support cell infiltration but also responds to the pathological oxidative stress at the lesion site, releasing therapeutic agents (e.g., docosahexaenoic acid) to further alleviate inflammation and promote neurogenesis [144]. Similarly, to solve the challenge of achieving injectability and post-implantation mechanical stability (a key requirement for the treatment of irregular lesions), researchers have developed innovative materials [145]. These gels exhibit low viscosity during injection for smooth delivery and rapidly form a robust, supportive network in situ, demonstrating how material microstructure can be engineered to meet complex clinical requirements. The most sophisticated designs integrate biochemical cues, such as the “cocktail hydrogel” enriched with neuroregulatory factors [146]. This system precisely controls nanostructures like pore size and stiffness to mimic the native brain ECM, significantly enhancing the survival, specific differentiation (e.g., into cortical interneurons), and functional integration of transplanted human neural progenitor cells for brain injury therapy [146].

#### 4.1.2. Pharmacological Targeting of Mechanosensory Pathways

Apart from biomaterial approaches, researchers also use drugs to block mechanotransduction pathways activated by abnormal stiffness. A key target is the mechanosensitive ion channel PIEZO1 [147]. Inhibitors such as Grammostola spatulata mechanotoxin 4 (GsMTx4) can effectively block stiffness-induced calcium influx in microglia, suppressing their pro-inflammatory polarization [49,148,149]. This strategy has shown promise in ameliorating neuroinflammation and cognitive deficits in models of Alzheimer’s disease. Recent research underscores the fundamental role of PIEZO1 and ECM-mechanotransduction in neural wiring itself. Studies using collagen—Imatrices have demonstrated that neurons sense and remodel the ECM through forces transmitted by dynamic collagen fiber bundles, and this mechanical communication—essential for precise neuronal connectivity and network formation-is highly dependent on PIEZO1 function [48,49]. These findings suggest that therapeutic mechanomodulation targets processes fundamental to both development and repair.

#### 4.1.3. Harnessing and Manipulating Dynamic Mechanical Signals

Emerging paradigms move beyond static stiffness modulation to actively program beneficial mechanical signals. A groundbreaking approach involves using magnetic manipulation to create dynamic matrix vibrations. One platform integrates Fe3O4 magnetic nanoparticles into a bioactive hydrogel, which, under a remote pulsed magnetic field, generates controlled stiffness oscillations within a physiologically relevant range [150]. This illustrates the potential of on-demand, reversible mechanical stimulation to guide cell fate. Similarly, for spinal cord injury repair, the concept of dynamic bioactive matrices has been leveraged in 3D bioprinting. Bioinks incorporating reversible chemical bonds (e.g., Schiff base) and bioactive peptides create a living construct where neural stem cells (NSCs) actively sense and remodel their matrix [151]. This enhanced cell–matrix interaction promotes NSC migration, neural network self-organization within the printed structure, and ultimately significant functional recovery in animal models. These advances highlight a shift from viewing the ECM as a static scaffold to treating it as a dynamic, instructive component that can be actively programmed to orchestrate regeneration.

Modulating ECM mechanical properties presents a powerful route to reprogramming the pathological glial and neural response. While soft hydrogels provide a permissive baseline, the future lies in fourth-generation smart materials that are injectable, dynamically responsive (to ROS, enzymes, or magnetic fields), and capable of delivering both biophysical and biochemical cues in a spatiotemporally controlled manner. The convergence of advanced biomaterials with a deeper understanding of mechanotransduction pathways like PIEZO1 is enabling unprecedented control over the neural repair microenvironment. The ultimate goal is to develop integrated systems that not only normalize static stiffness but also deliver therapeutic dynamic mechanical signals, thereby synergistically with cellular and molecular strategies to rebuild functional neural tissue.

### 4.2. Modulating ECM Composition

Building on the understanding that specific ECM molecules critically influence glial cell behavior and neural regeneration, therapeutic strategies have been developed to directly target the aberrant biochemical landscape of the pathological neural microenvironment. These approaches can be broadly categorized into two complementary paradigms: the removal of inhibitory molecules and the restoration or supplementation of beneficial ones, with recent advances focusing on engineering these interventions for greater precision and efficacy.

#### 4.2.1. Degradation of Inhibitory ECM Components: From Proof-of-Concept to Precision Delivery

Strategies to break down CSPG-derived inhibitory barriers have moved from basic validation to optimized delivery systems addressing delivery and stability. Intrathecal delivery of chondroitinase ABC (ChABC) has been extensively validated in preclinical models for its ability to promote axonal sprouting, enhance synaptic plasticity, and improve functional recovery after CNS injury [152,153]. The current research frontier focuses on overcoming the inherent instability of the native enzyme and achieving its sustained, controlled release at the lesion site. For instance, cutting-edge protein engineering has led to the development of a ChABC variant with 37 point mutations (ChASE37), which exhibits significantly enhanced thermostability [154]. To enable its precise delivery to the brain, this engineered enzyme has been formulated as a fusion protein and integrated with a chemically modified, injectable carboxymethyl cellulose hydrogel [155]. This “affinity-release” hydrogel system enables the localized and prolonged delivery of ChASE37 to a rat stroke lesion, effectively degrading CSPGs and inducing axonal sprouting within and around the infarct core, thereby laying a foundation for tissue regeneration [155]. Such smart delivery systems represent a critical direction for translating enzymatic therapy into a clinically viable strategy.

#### 4.2.2. Regulating Hyaluronan Dynamics: Harnessing Its Dual Nature

Therapeutic strategies targeting HA exploit its molecular-weight-dependent “dual nature.” On one hand, supplementation with HMW-HA can exert direct anti-inflammatory effects by suppressing microglial activation via TLR2/4 receptors [156]. On the other hand, more sophisticated interventions target pro-inflammatory, low-molecular-weight HA fragments that accumulate after injury. For example, using HA oligosaccharides to competitively disrupt pathological HA-protein complexes (e.g., with heavy chains) has been shown to reduce CD44/TLR4 expression and dampen pro-inflammatory cytokine release [157]. An even more innovative approach involves designing novel HA-based biomaterials to actively create a pro-regenerative niche. One study developed a hyaluronan-silk fibroin co-crosslinked hydrogel (HA-SF gel) [158]. This material not only serves as a long-lasting physical scaffold but also significantly promotes the secretion and remodeling of type III collagen (a “gold-standard” collagen) by activating dermal fibroblasts [158]. This suggests that through functional design and composite engineering, HA-based materials can evolve from passive regulators to active inducers of tissue repair.

#### 4.2.3. Supplementation and Engineering of Protective ECM Molecules: From Replacement to Mimicry

Beyond removing inhibitors, actively constructing a supportive matrix network is equally crucial. Supplementing key adhesive glycoproteins, such as laminin and fibronectin, provides essential signals for cell survival, adhesion, and migration [159,160]. This concept has been extended to the more advanced frontier of biomaterial engineering. Recent studies show that by designing proteoglycan mimetics capable of binding specific cell surface receptors (e.g., αvβ3 integrin), the secretory function of resident cells can be precisely modulated. For instance, a collagen-binding proteoglycan mimetic (LDS) was shown to significantly alter the proteome of extracellular vesicles secreted by endothelial progenitor cells, enriching factors related to angiogenesis and thereby functionally enhancing their pro-angiogenic capacity [161]. This provides a novel paradigm for developing next-generation ECM-mimetic materials that can “educate” host cells to shift their secretome towards a reparative phenotype.

Maintaining appropriate levels of FN and LN after injury can support the survival and phagocytic function of microglia, maintain a high number of glial cells, reduce neuronal injury, and provide necessary adhesion support for neurons, reducing injury [82]. Components such as FN can also maintain the content of collagen and increase the number of functional neurons [82].

### 4.3. Reprogramming Glial Phenotypes

Beyond modulating the ECM itself, directly targeting glial cell phenotypes to steer their functional state represents another pivotal therapeutic axis. This strategy aims to suppress detrimental pro-inflammatory activities while promoting reparative functions, thereby reshaping the neural microenvironment at the cellular level.

#### 4.3.1. Targeting Extracellular Proteases and Their Inhibitors

Many studies aim to restore the balance between matrix metalloproteinases (MMPs) and their endogenous inhibitors, such as tissue inhibitor of metalloproteinases-1. While broad-spectrum MMP inhibitors have shown benefits in improving motor recovery after spinal cord injury by curbing excessive pathological ECM degradation, their application requires precise control to preserve necessary ECM remodeling for repair [162]. TIMP-1, as a major endogenous inhibitor of MMP-9, plays a multifaceted role in maintaining ECM and neurovascular unit homeostasis [163]. It regulates processes including astrocyte proliferation, neural stem cell adhesion/migration, and oligodendrocyte differentiation, primarily through activating FAK/phosphoinositide 3-kinase (PI3K)-Akt and MAPK signaling pathways [163]. Furthermore, TIMP-1 exerts indirect immunomodulatory effects by influencing microglial chemotaxis and IL-1β signaling, and directly protects neurons by inhibiting aberrant calcium influx and Aβ toxicity via the LRP-1 receptor [163]. However, the therapeutic window for modulating this system is narrow, as both excessive protease activity and over-inhibition can impede recovery.

#### 4.3.2. Modulating Astrocyte Reactivity via ECM Cues

Another promising strategy is to exploit native ECM molecules that naturally regulate glial states. For instance, Tenascin-C (TN-C) has been shown to reverse the activation phenotype of reactive astrocytes, promoting their return to a more quiescent state [164]. This effect, which is partially reversible, positions TN-C and its signaling pathways as a potential target for mitigating glial scar formation and its associated inhibition of regeneration.

In summary, glial phenotype reprogramming-via modulating protease equilibrium or ECM-dependent signaling-can normalize cellular dysfunction in pathological neural microenvironments. When combined with approaches targeting ECM mechanics and composition, these strategies constitute an integrated therapeutic framework for neurological disorders.

## 5. Summary, Open Questions and Future Perspectives

### 5.1. Current Research Achievements

Research on glia-ECM interactions has achieved substantial progress. Researchers have identified four major ECM subtypes, and defined unified stiffness values for physiological and pathological conditions. A full set of tools (AFM, MRE, rheometer) is now available for CNS mechanical testing and intervention.

It is well verified that ECM transmits both mechanical (PIEZO1, integrin, NMII) and biochemical signals to regulate glial phenotypes. In return, astrocytes, microglia and oligodendrocyte lineage cells remodel ECM via synthesis, degradation and phagocytosis, forming a dynamic bidirectional regulatory network. In addition, three categories of interventions (modulating ECM mechanics, optimizing matrix composition, reprogramming glial phenotypes) have shown promising therapeutic effects in preclinical studies.

### 5.2. Key Open Questions and Existing Bottlenecks

Current studies mainly focus on individual axes of mechanotransduction, biochemical signaling and ECM remodeling, that fails to reveal the holistic spatiotemporal network in native CNS tissues. Most in vitro models cannot recapitulate the complex crosstalk among neurons, multiple glial subsets and dual mechanical-biochemical signals. Current detection methods still have drawbacks. AFM cannot perform long-term in vivo monitoring, and MRE lacks microscale resolution for subtle lesions.

In addition, current drugs and biomaterials suffer from insufficient specificity and stability. Large discrepancies between rodent and human neural tissues create a prominent translational gap. The fine functions of specialized ECM structures such as PNN and perinodal ECM remain poorly understood, and synergistic combinations of multiple therapeutic strategies have not been systematically explored.

### 5.3. Future Research Directions

To solve these bottlenecks, interdisciplinary technologies will dominate future research. Spatial transcriptomics and single-cell proteomics enable high-resolution profiling of glia and ECM. Engineered biomaterials and organ-on-a-chip systems also help dissect complex physical and chemical cues in vitro. Computational modeling and artificial intelligence will be applied to analyze high-dimensional datasets, predict network dynamics and identify therapeutic targets.

From the therapeutic perspective, the three intervention strategies are mutually complementary rather than exclusive. The next research frontier lies in rationally combining mechanical modulation, ECM composition regulation and glial phenotype reprogramming, based on systematic understanding of the pathological microenvironment. Optimizing glia-ECM crosstalk and glial phenotypes will be the core to achieving effective neural repair.

### 5.4. Concluding Remarks

The glia-ECM mechanochemical axis is a core regulator of CNS homeostasis and diseases. While many challenges remain, the combination of mechanobiology, biomaterials and multi-omics will advance this field, and may yield new therapies for hard-to-treat neurological diseases.

## Figures and Tables

**Table 1:** Composition and functional characteristics of major ECM subtypes in the CNS.

ECM Subtype	Major Molecular Composition	Structural Features	Core Functions
Interstitial ECM	HA, Lectican family, CSPGs, Tenascins	Amorphous network filling extracellular spaces	Ion buffering, neurotransmitter diffusion regulation, hydration
Perineuronal Nets (PNNs)	CSPGs, Aggrecan, Tenascin-R, HA, HAPLN1/Crtl1	Reticular coating on neuronal surfaces	Synaptic stabilization, excitation/inhibition balance, critical period plasticity control
Perinodal ECM	Tenascin-R, Versican, HA HAPLN2/Bral1	Specific wrapping around axon nodes of Ranvier	Sodium channel anchoring, action potential conduction
Basement Membrane	Collagen IV, Laminin, Fibronectin, Perlecan, Nidogen	Continuous thin layer between endothelial cells and parenchyma	Blood–brain barrier, cell polarity regulation, ion channel anchoring

**Table 2:** Regulatory effects of major ECM components on glial cells.

ECM Component	Receptors	Astrocytes	Microglia	Oligodendrocytes
Laminin	Integrin β1, Dystroglycan	Mediates Kir4.1/AQP4 clustering, regulates ion homeostasis [71,75,78]	Weak adhesion via α6β1 integrin, PKC-dependent activation [18]	Promotes OPC migration, differentiation, myelination via integrin β1-FAK [88,89]
Fibronectin	Integrin α5β1	-	Promotes adhesion proinflammatory activation [18]	Early: supports OPC recruitment; late: inhibits differentiation [90]
HA	TLR2/4, CD44	HMW-HA maintains quiescence; LMW-HA promotes reactivity [57]	LMW-HA drives proinflammatory activation via TLR2/4 [23]	High concentrations inhibit OPC differentiation and remyelination [91]
CSPGs	PTPσ	Form PNN ion-buffering structure; over-secretion creates inhibitory scar [24]	Induces activation, proliferation, IGF-1, MMP-2/9 expression [23]	
Tenascin-C	Integrins, FnIII domains	Supports migration/proliferation of reactive astrocyte subsets during scar formation [92].	Modulates inflammatory responses [93]; context-dependent.	Inhibits OPC migration [94], promote differentiation via intracellular pathways [29,95]
Tenascin-R	Contactin, F3/11	Contributes to PNN stability; modulates excitability and plasticity [96]	modulate inflammatory signaling [97].	Stabilizes axon-OL contact and induces myelin-specific gene expression [86].

## Data Availability

No new data were created or analyzed in this study. Data sharing is not applicable to this article.

## Abbreviations

The following abbreviations are used in this manuscript:

AD	Alzheimer’s disease
ADAMTS	A Disintegrin And Metalloproteinase with Thrombospondin Motifs
AQP4	Aquaporin-4
BBB	Blood–Brain Barrier
CNS	Central Nervous System
CSPGs	Chondroitin Sulfate Proteoglycans
ECM	Extracellular Matrix
FAK	Focal Adhesion Kinase
FN	Fibronectin
GLT-1	Glutamate Transporter 1
HA	Hyaluronan
HMW-HA	High-Molecular-Weight Hyaluronan
IGF-1	Insulin-like Growth Factor 1
IL-1β	Interleukin-1β
ILK	Integrin-Linked Kinase
Kir4.1	Inwardly Rectifying Potassium Channel 4.1
LMW-HA	Low-Molecular-Weight Hyaluronan
LN	Laminin
MMPs	Matrix Metalloproteinases
MRE	Magnetic Resonance Elastography
NMII	Non-muscle Myosin II
OPCs	Oligodendrocyte Precursor Cells
PIEZO1	Piezo type mechanosensitive ion channel component 1
PKC	Protein Kinase C
PNNs	Perineuronal Nets
ROS	Reactive Oxygen Species
TIMP	Tissue Inhibitor of Metalloproteinases
TLR2/4	Toll-like Receptors 2 and 4
TN-C	Tenascin-C
TN-R	Tenascin-R
TNF-α	Tumor Necrosis Factor-α
tPA	Tissue Plasminogen Activator
